# Microfluidic Point-of-Care Testing: Commercial Landscape and Future Directions

**DOI:** 10.3389/fbioe.2020.602659

**Published:** 2021-01-15

**Authors:** Shivangi Sachdeva, Ronald W. Davis, Amit K. Saha

**Affiliations:** Genome Technology Center, School of Medicine, Stanford University, Palo Alto, CA, United States

**Keywords:** point-of-care diagnostics, LFA, NAATs, COVID-19 diagnostics, microfluidics

## Abstract

Point-of-care testing (POCT) allows physicians to detect and diagnose diseases at or near the patient site, faster than conventional lab-based testing. The importance of POCT is considerably amplified in the trying times of the COVID-19 pandemic. Numerous point-of-care tests and diagnostic devices are available in the market including, but not limited to, glucose monitoring, pregnancy and infertility testing, infectious disease testing, cholesterol testing and cardiac markers. Integrating microfluidics in POCT allows fluid manipulation and detection in a singular device with minimal sample requirements. This review presents an overview of two technologies - (a.) Lateral Flow Assay (LFA) and (b.) Nucleic Acid Amplification - upon which a large chunk of microfluidic POCT diagnostics is based, some of their applications, and commercially available products. Apart from this, we also delve into other microfluidic-based diagnostics that currently dominate the *in-vitro* diagnostic (IVD) market, current testing landscape for COVID-19 and prospects of microfluidics in next generation diagnostics.

## Introduction

Innovation in technology and emerging at-home diagnostic tests have played an important role in early disease detection, diagnosis and maintenance. “Point-of-care testing” (POCT) - Tests at or near the patient site was a term introduced in early 1980's but such a system was first developed in 1972 (Gerald et al., 2014). Over the last decade, POCT has been widely accepted as a rapid and an economical form of diagnostic tool as compared to traditional laboratory-based testing especially in low resource settings (Pai et al., 2012).

**Figure 1:**
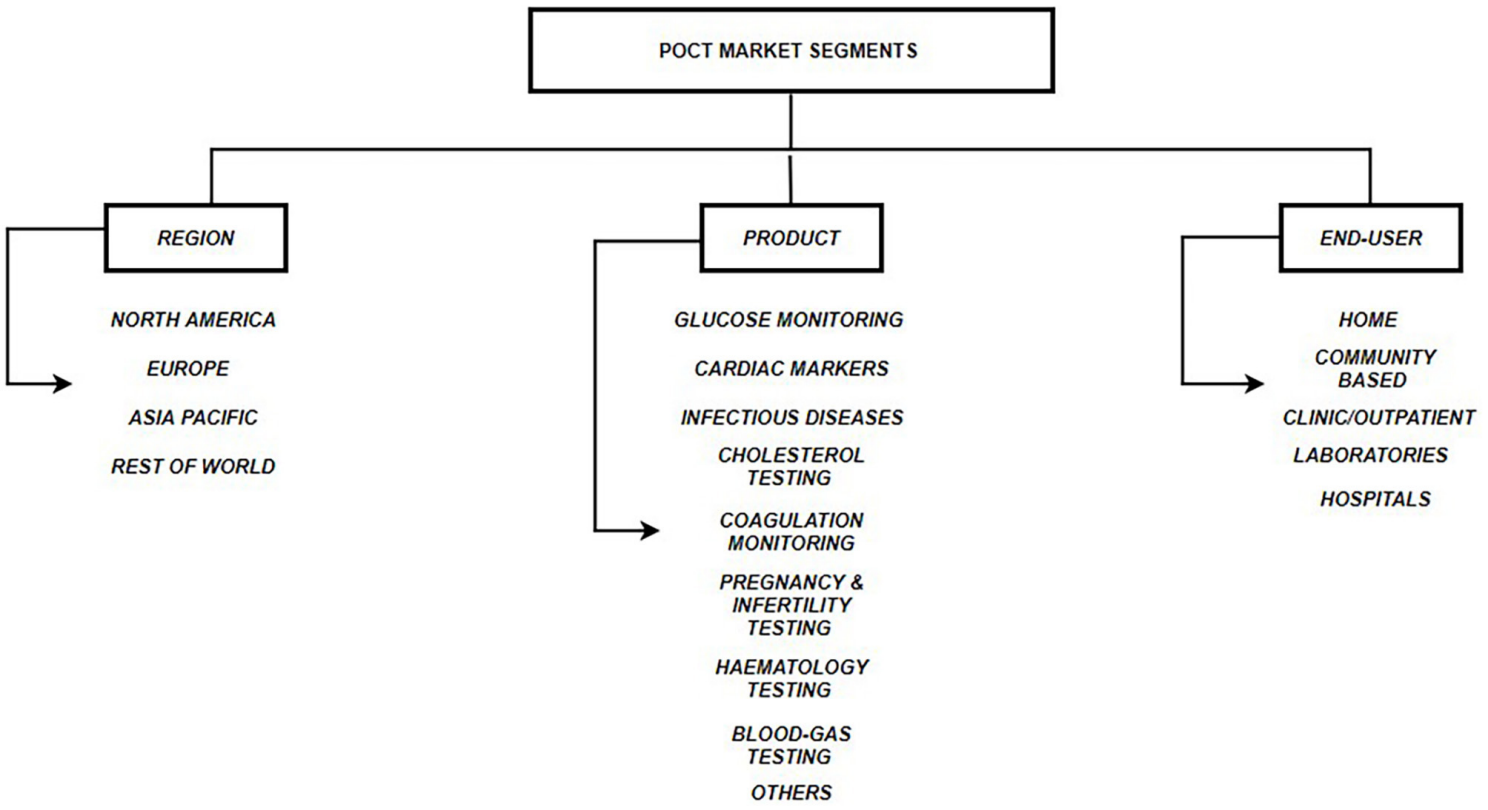
POCT Market Segments. Three segments of the global POCT market include: (a.) Regional - North America, Europe, Asia Pacific & Rest of World; (b.) Product Type - Glucose monitoring, Cardiac markers, Infectious disease testing, Cholesterol testing, Coagulation monitoring, Pregnancy & Infertility testing, Hematology testing, Blood-gas testing, Others; (c.) End User - TPP1 - Homes, TPP2 - Communities, TPP3 - Clinics, TPP4 - External Laboratories, TPP5 – Hospitals (Pai et al., 2012; DUBLIN, 2018; Kaur et al., 2019).

**Figure 2:**
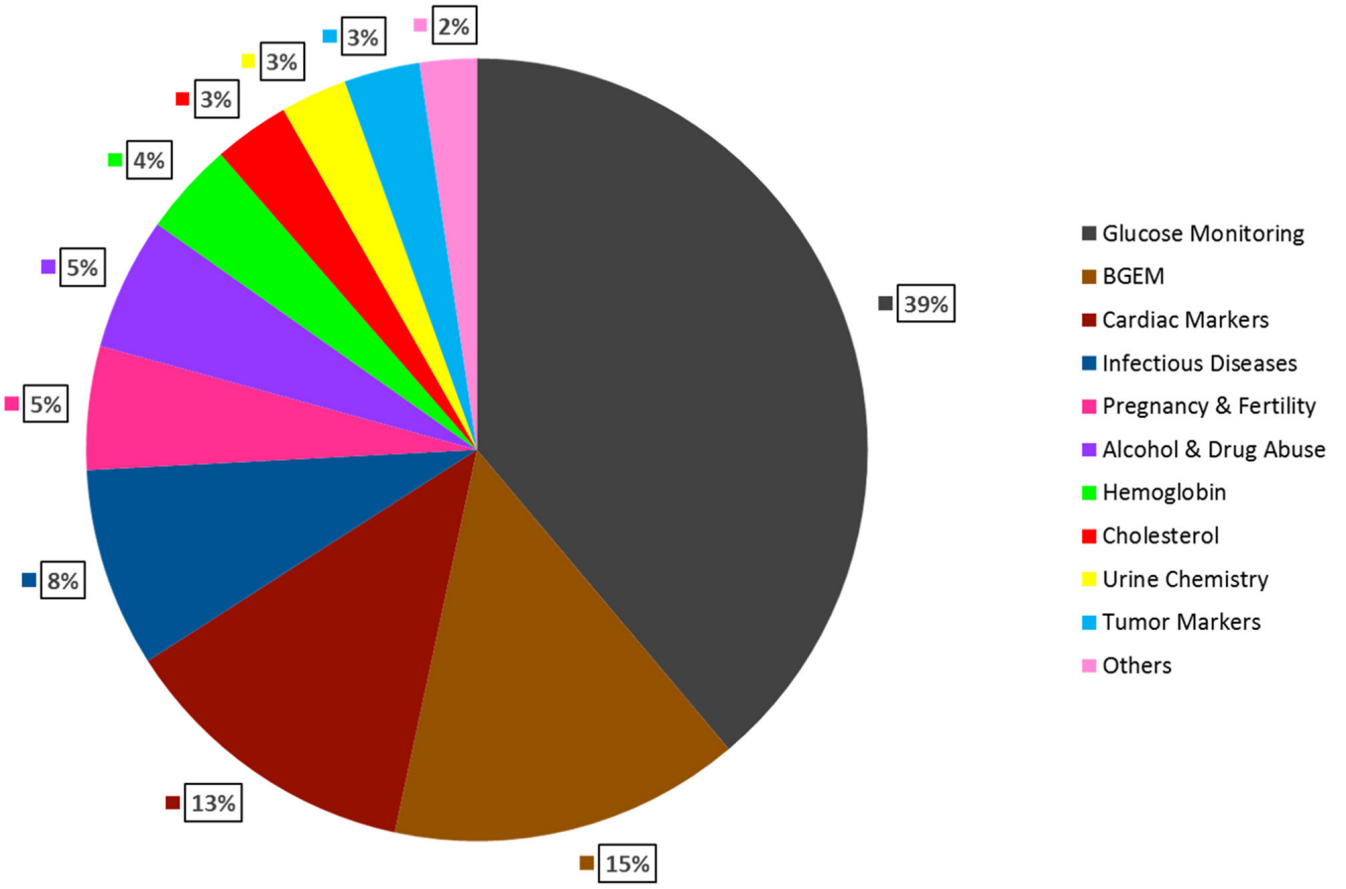
Global Anticipated Revenue Generation by Product Type in 2022. Glucose Monitoring is expected to have the largest market share (39%), followed by, Blood gas testing (15%), Cardiac markers (13%), Infectious Diseases (8%), Pregnancy & Fertility testing (5%), Alcohol & Drug Abuse (5%), Hemoglobin testing (4%), Cholesterol testing (3%), Urine chemistry (3%), Tumor markers (3%), Others (2%) (DUBLIN, 2018).

Other advantages of POCT include - (a.) Simple to operate; (b.) No need for trained professionals; (c.) Cost-effective; (d.) Easy to fabricate in bulk; (e.) Rapid turn-around-time (Luppa et al., 2011). POCT is shown to be of utmost importance in situations where rapid medical decisions need to be taken for example in emergency departments. Several studies have shown that use of POCT diagnostics reduce overall per patient cost, length of stay in hospitals and provide faster results as compared to a traditional laboratory testing. Quality assessment and satisfaction surveys conducted on medical staff and physicians have also shown positive affirmations toward the use of POCT. Therefore, POCT is an important tool, which can reduce mortality, morbidity and improve the quality of life (Lee-Lewandrowski et al., 2003; Kankaanpää et al., 2018). Figure 1 describes the three segments that make the POCT market: Region, Product and End-user. Figure 2 describes the revenue that the POCT market is anticipated to generate in 2022.

To assist in the development of diagnostics in low resource settings, The World Health Organization (WHO) has put forward a set of guidelines referred to as “ASSURED” - (a) affordable, (b) sensitive, (c) specific, (d) user friendly, (e) rapid and robust, (f) equipment-free, and (g) deliverable to end-users (Urdea et al., 2006; Su et al., 2015; Tay et al., 2016). These guidelines seem to be compatible with the emerging microfluidic technologies. Microfluidic based POCT devices are widely used for molecular biology, chemical and biochemical analysis. Microfluidic technology enables detection and fluid regulation in one single component; increased sensitivity and specificity to detect target analytes at small volumes overcomes several challenges encountered while using traditional POCT diagnostics (Pandey et al., 2018).

In this review, we discuss various commercially available point-of-care diagnostics and their underlying technology.

## Commonly Used Fabrication Techniques For POCT Devices

### Paper Based Devices/μPADS

Using filter paper and paraffin, the concept of microfluidic channels on paper was first introduced by Müller and Clegg (1949). However, the first functional paper device which could perform a protein glucose assay was created by the Whitesides' Group at Harvard University in 2007 (Martinez et al., 2007). Due to its biocompatible properties with various substrates, lightweight, flexibility, low cost, hydrophilic nature, ease of use and availability, paper has become a popular substrate for microfluidic applications such as dipstick tests, Lateral Flow Assays (LFAs) and microfluidic analytical devices (μPADS) (Akyazi et al., 2018). Raw materials such as linen, jute, hemp, bamboo, sisal, grass, wood, cotton and straw are used to make paper (Yetisen et al., 2013). These sources make paper fibrous and porous due to which paper as a substrate is - (a.) Absorbent: to store liquid and allow movement of liquid through capillary action; (b.) Air permeable: to act as a filter; (c.) Has high surface-volume ratio: to immobilize high amounts of liquid (Wang et al., 2012; Akyazi et al., 2018). Cellulose and Nitrocellulose are two key materials used in point-of-care testing devices. Filter and Chromatography paper derived from cellulose are used to make dipsticks and PADS. Whereas, Nitrocellulose is used to make LFAs (Yetisen et al., 2013).

Two commonly used fabrication techniques for paper-based devices include - Cutting and Hydrophobic/ Hydrophilic Contrast. In cutting technique, a computerized cutter plotter or a carbon dioxide laser cutting apparatus is used to create channels on the paper. These channels are then covered with sticky tape (Akyazi et al., 2018). Due to need for specialized equipment and the high fabrication cost, this fabrication method is not widely used. Whereas, in the latter, hydrophobic patterning agents such as photoresist SU-8, wax and alkyl ketene dimer (AKD) are used to create hydrophilic channels on the paper. Patterning of channels can be done in three ways - (a.) Physical blocking of pores on paper using PDMS or photoresist; (b.) Physical deposition of agents on the cellulose fibers using polystyrene or wax; (c.) Chemically modifying the paper fibers with cellulose reactive agents like AKD (Li et al., 2012). Various other fabrication techniques for paper devices include – photolithography, ink-jet printing, PDMS plotting, wax printing, wax dipping, wax screen printing, plasma treatment, ink-jet etching, flexography printing, stamping, wet etching and cutting (Akyazi et al., 2018).

### Polymer Based Devices

The techniques to fabricate these devices were inspired from the semiconductor industry and microelectronic. Glass and Silicon were the first substrates used and channels could either be etched or created using photolithography. However, since both these materials are expensive, plastic and polymers are now being used as alternatives (McDonald et al., 2000; Qamar and Shamsi, 2020).

Polyethylene naphthalate (PEN), Polyethylene terephthalate (PET), Polyimide (PI), Polyetheretherketone, Polyethersulfone (PES) and Polycarbonate are plastic substrates that are stable at high temperatures (Weltin et al., 2014; Chen et al., 2015; Kokkinos et al., 2015; Saha et al., 2017a; Tur-García et al., 2017). Wax-on-plastic (Wax printing) is a novel technique used to create microchannels on Polyethylene terephthalate (PET) (Qamar et al., 2019).

Polymers such as Polytetrafluoroethylene (PTFE), Poly (methyl methacrylate) (PMMA), Polydimethylsiloxane (PDMS) are also being used as substrates to fabricate these devices. Polymers are inexpensive and microchannels are created by embossing and molding (Saha et al., 2017b, 2019). Due to low autofluorescence, biocompatibility, non-toxicity, curing at low temperatures, PDMS is the most commonly used polymer. Chips can be fabricated using techniques such as soft lithography, Hot embossing, Injection molding, UV-printing, Laser Photoablation and 3D-Printing (Kiran and Chakraborty, 2020).

### Textile Based Devices

Although paper and PDMS are the most commonly used substrates; textiles such as threads, fibers and fabrics have recently been considered as alternatives to fabricate microfluidic devices (Naeimirad et al., 2019; Rumaner et al., 2019). “Lab-on-fiber” technology is suitable for diagnostics in low resource settings, as they are low-cost and need very small volumes of samples (Ricciardi et al., 2015). Several other advantages include high flexibility, ability to be designed into wearables, high strength, no requirement of hydrophilic-hydrophobic contrast barrier, 3D structure, automatic transport without the need of external pumps and biocompatibility (Nilghaz et al., 2013; Caetano et al., 2018; Naeimirad et al., 2019). In lab-on fiber, threads act as microchannels, hence it eliminates the additional fabrication steps (Ricciardi et al., 2015). Since threads can transport liquids through capillary pressure and wicking, it is important to choose the right fiber type for weaving (Naeimirad et al., 2019). Surface treatment of natural and synthetic fibers can enhance the wicking abilities by removing the hydrophobic wax (Xing et al., 2013). Silk (Bhandari et al., 2011), Polyester and Cotton have recently been used as microfluidic platforms (Reches et al., 2010; Safavieh et al., 2011). Manipulation of fluid flow and mixing was also demonstrated through knots and thread weaving (Li et al., 2010).

## Commonly Employed POCT Technologies

**Figure 3:**
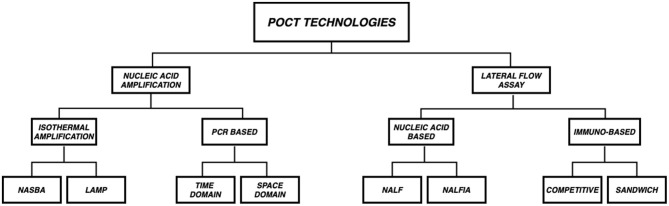
Overview of POCT Technologies. POCT platforms are mostly based on two technologies (a.) Lateral Flow Assay (LFA) and (b.) Nucleic Acid Amplification (NAAT). LFAs are subclassified into Immuno based (Competitive and Sandwich) and Nucleic acid based [Nucleic Acid LFA (NALF) and Nucleic Acid Lateral Flow Immunoassays (NALFIA)] whereas NAATs are subclassified as PCR (Time Domain and Space Domain) and Isothermal amplification (NASBA and LAMP) based.

In this section we discuss the two most common technologies upon which microfluidic POCT diagnostics are based - (a.) Lateral Flow Assay (LFA) and (b.) Nucleic Acid Amplification. Figure 3 gives a schematic overview of the different subtypes in the aforementioned technologies.

### Lateral Flow Assay Technology

#### Introduction

The technical principle of this assay was derived from lateral fixation tests, which were used to diagnose rheumatoid arthritis in 1956 (Singer and Plotz, 1956). LFA technology was first observed in the late 1960's to detect proteins in serum. A homemade test to detect human chorionic Gonadotropin in urine was the first of its kind to be developed in 1976 (Bahadir and Sezgintürk, 2016).

The most frequently used lateral flow strips are paper based (Estrela et al., 2016). Different types of biological fluids such as plasma, blood (Schramm et al., 2015), urine (Moreno et al., 2017), saliva (Carrio et al., 2015), and serum can be used as samples (Magambo et al., 2014). Broadly, LFA tests can be divided into two categories:

(a.) Lateral flow immunoassay tests, where antibodies are used to recognize antigens, proteins or hormones. These assays are classified into two types: (1.) Sandwich LFA and (2.) Competitive LFA (Estrela et al., 2016).

(b.) Lateral flow nucleic acid tests, where DNA/RNA oligonucleotides or synthetically produced short single stranded DNA/RNA sequences called aptamers are used as biorecognition elements. These assays can be grouped into two categories (1.) Nucleic Acid LFA (NALF) and (2.) Nucleic Acid Lateral Flow Immunoassays (NALFIA) (Estrela et al., 2016; Wang et al., 2018).

#### Lateral Flow Strip Design

A LFA strip usually consists of the following components:

- Sample pad. The first section of the strip where the pipetted biological sample gets absorbed. This pad may be treated with certain salts or surfactants to maintain the pH or control the flow rate of the sample (Estrela et al., 2016).
- Conjugate pad. It consists of immobilized pre-labeled sample recognition elements. Different types of reporter labels - gold nanoparticles, fluorescence quenching labels, quantum dots can be used (Estrela et al., 2016).
- Detection zone, a porous layer usually made of nitrocellulose membrane that allows interaction of antigen and antibody or DNA/RNA hybridization. This region contains the test line and the control line. Wherein, the antibody or nucleic acid hybrid specific to the sample is immobilized on the test line and the control line consists of a secondary sample recognition element. We can add several lines in this region of the strip to check for different analytes from the same sample simultaneously (Xu et al., 2014; Yen et al., 2015).
- Absorption pad. Typically the last section of the lateral flow strip. This collects excess waste and ensures that there is no back flow of the fluid (Estrela et al., 2016).

**Figure 4:**
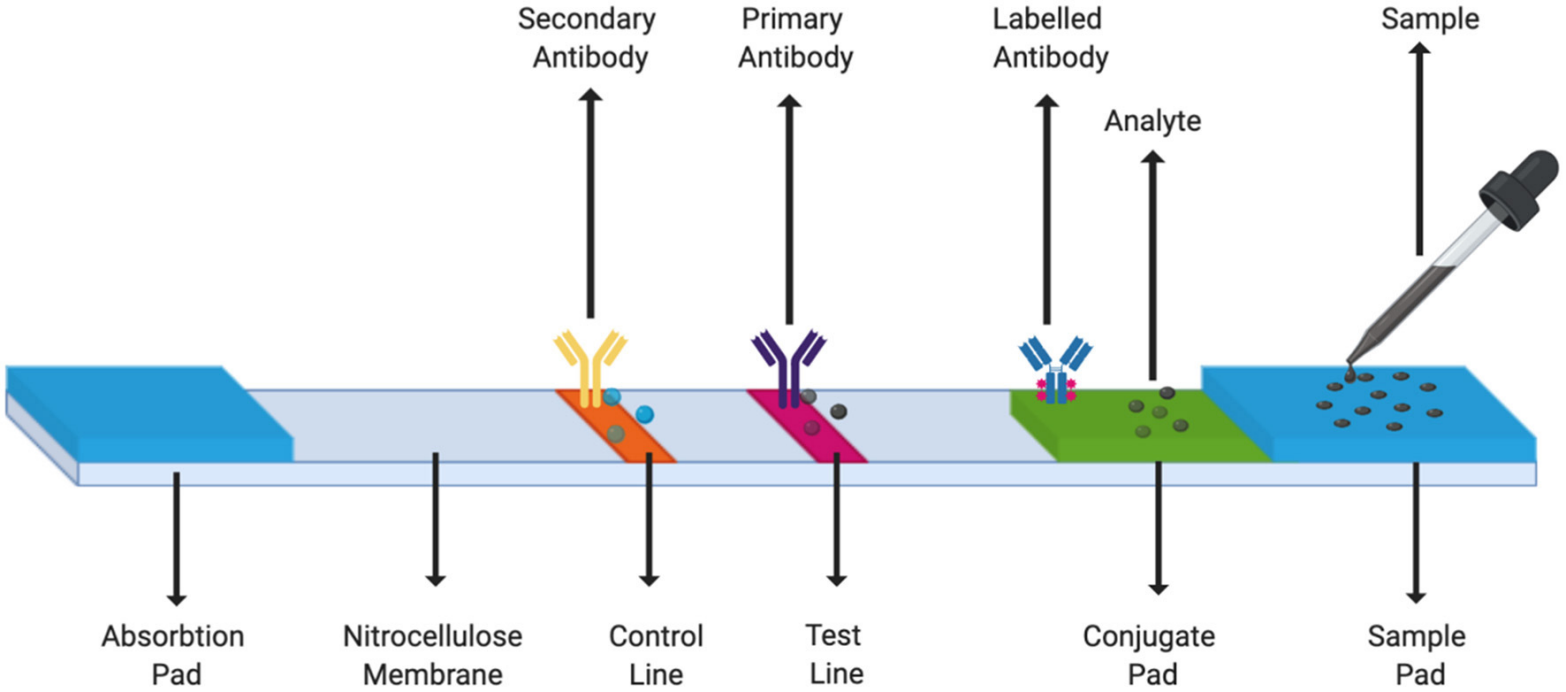
LFA Strip Components. A simple LFA test strip consists of a sample pad, a conjugate pad, test line, control line, and an absorption pad.

Figure 4 is a schematic of a typical LFA strip. The sample containing the target analyte is absorbed by the sample pad and it moves toward the conjugate pad with the help of capillary forces. Here, the analyte interacts with a specific antibody or DNA/RNA oligonucleotide (labeled with a colored molecule) and forms a mobile conjugate which flows onto the nitrocellulose membrane. The conjugates which are complementary to the immobilized bioreceptors on the test and the control lines, get captured, respectively, whereas, the remaining fluid gets wicked by the absorbent pad. A signal is generated on the lines as soon as the conjugate containing the reporter label along with the target analyte binds to its bioreceptor. As a result, a change in the color of the lines can then be seen (Mahmoudi et al., 2020).

#### Applications and Commercially Available Products

LFAs can be used for diagnosis and prognosis of diseases like cancer by identifying specific biomarkers. Researchers have identified several biomarkers in cancer - DNA/RNA sequences, enzymes, small molecules, proteins, hormones, extracellular vesicles and circulating tumor cells (CTC), which adapt either the sandwich format or competitive format of LFAs (Mahmoudi et al., 2020).

LFA strips are widely being used in point-of-care testing (POCT). At home pregnancy tests developed by Ept was the first of its kind to be available commercially in 1976 (Yen et al., 2015). However, this technology has evolved over time with digital pregnancy tests as the next generation tests. Clear Blue Digital Pregnancy Test was the first FDA approved digitized test to have been launched in the market in 2003 (Etherington, 2020). Lateral flow technology is also in use for rapid HIV testing; Visitect CD4 is an example of a rapid disposable LFA test developed for HIV patients, it quantifies CD4 protein in T-cells (Ndlovu et al., 2020). Other examples of POCT technologies include - ProFlow, C.Diff Quik Chek & Sickle SCAN. ProFlow is a rapid test which detects Glutamate Dehydrogenase (GDH) in human feces to diagnose Clostridium difficile (C. difficile) infection (Pezzuto et al., 2019). C. diff Quik Chek is another enzyme linked immunoassay that uses glutamate dehydrogenase specific antibodies of *C. difficile* to detect GDH, Toxins A and B in fecal samples. It can provide results within 30 min (Health Care, 2020). Sickle SCAN at home test determines sickle cell trait and aids in diagnosis of Sickle cell disease (POCT Market Forecast, 2020). Alere Determine HIV-1/2 Ag/Ab by Abbott Diagnostics is the first FDA approved, CLIA waived LFA-based finger stick test, it can detect HIV-1/2 antibodies and free HIV-1 p24 antigen within 20–30 min (Kanter et al., 2015). Other disorders which rely on LFAs in diagnostics include - bloodborne diseases such as Herpes Simplex Virus, Hepatitis A, B, C; Infectious diseases such as Ebola, Dengue, Malaria, Zike; Sexually transmitted infections; respiratory infections (de Puig et al., 2017; Hristov et al., 2019).

Lateral flow technology has also been used in detection of various bioterrorism pathogens and toxins such as *Bacillus anthracis* (Cox et al., 2015), *Francisella tularemia* (Pillai et al., 2020)*, Yersinia pestis* (Hsu et al., 2018), *Clostridium botulinum* (Gessler et al., 2007), and *Staphylococcal* enterotoxin B (Parida et al., 2020).

#### Advantages

LFA tests have completely satisfied the ASSURED criteria established by the World Health Organization (WHO). It is the only diagnostic platform that covers the wide range of target product profile (TPP) from level 1 to level 5 (Reid et al., 2020). This technology has shown consistent reproducibility & specificity with rapid outcomes. Due to low production cost, portability, ease of use and long shelf life, these tests are suitable for use in developing & low resource countries (Kanter et al., 2015; Mahmoudi et al., 2020). Depending upon the test, a variety of body fluids can be used as samples at small volumes and analysis of the results does not require additional instruments or expertise making this technology easily accessible (Estrela et al., 2016).

#### Limitations/Challenges

Some of the limitations of/challenges with using LFA tests, are: (a.) Limitation in sensitivity (Estrela et al., 2016; Yew et al., 2018; Mahmoudi et al., 2019); (b.) Accuracy of the test relies on the quality and preparation of the antibodies; (c.) Analysis time is dependent on the physical properties of the sample, for example, if the sample is too viscous, analysis may take longer (Estrela et al., 2016); (d.) The results obtained are qualitative or semi-quantitative, whereas some diseases require accurate quantitative results in order to make a correct decision in terms of disease diagnosis (Li and Macdonald, 2016; Hu et al., 2017; Yew et al., 2018); (e.) Additional equipment may be necessary (Yew et al., 2018); (f.) Since LFAs are dependent on immobilized proteins and antibodies as ligands, multiplexing assays poses a challenge due to lot-to-lot variability and antibody cross reactivity (Haushalter et al., 2016; Mohd Hanafiah et al., 2017).

### Nucleic Acid Amplification Technology

#### Introduction

Although LFAs have widely been used to detect an array of pathogens and proteins via antibody and nucleic acid amplification, it lacked sensitivity and specificity beyond a certain level, due to poor detection techniques (Tang et al., 2017; Yew et al., 2018). Hence, there was a need for new technology - Nucleic Acid Amplification Tests (NAATs). The working principle of these tests is to generate multiple copies of the pathogen's DNA/RNA sequence using a detection probe and to produce a signal; the amount of signal produced is directly proportional to nucleic acid concentration. Therefore, NAATs is a highly specific and a sensitive tool for disease diagnosis (Weigl et al., 2008; Ballard and Ozcan, 2018).

There are three steps in a NAAT assay: sample preparation, amplification, and detection. Sample preparation involves chemical or enzymatic lysis, nucleic acid purification followed by elution. Biological samples from blood, saliva, urine and tissue can be used as samples for detection (Chiappin et al., 2007; Sun et al., 2014). Amplification can be by two ways – (a.) Polymerase Chain Reaction (PCR) on a chip - A miniaturized version of the conventional PCR which requires three different temperatures to amplify (Niemz et al., 2011) or (b.) Isothermal Amplification – Using specific enzymes denature the double strands of DNA, it operates at low temperatures, consumes less power and does not require heavy equipment (Craw and Balachandran, 2012). Although several types of Isothermal Amplification techniques such as rolling circle amplification (RCA), nucleic acid sequence based amplification (NASBA), and strand displacement amplification (SDA) still require denaturation at high temperatures, other techniques such as recombinase polymerase amplification (RPA) and loop-mediated amplification (LAMP) do not use heat for denaturation of the strands (Tsaloglou et al., 2015; Lee et al., 2019). Detection can either be end-point or real-time [using different fluorescence-based dyes such as SYBR Green I or hydroxyl naphthol blue and instruments such as BART, Loopamp Realtime Turbidimeter (Niemz et al., 2011; Ding et al., 2015)].

#### Applications and Commercially Available Products

Loopamp is a detection kit developed by Eiken Chemical Co, Ltd which is based on LAMP isothermal amplification and can be used to detect an array of infectious diseases such as *Mycoplasma pneumonia, Bordetella pertussis, Legionella pneumonia, H1 pdm 2009 Influenza, Influenza A virus, Influenza A subtype H5, SARS, Legionella, Aspergillus, Herpes simplex*, and *West Nile Virus* (WNV) (Weigl et al., 2008). It is now also used to diagnose SARS-Cov-2 (Yan et al., 2020). Revogene and Alethia by Meridian Bioscience are rapid molecular diagnostic platforms. Revogene is a microfluidic cartridge based real time PCR device and it can test for *C. difficile, Strep B*, and *Streptococcus A* and provide results in about 2 min (Yan et al., 2020). Alethia is a LAMP based diagnostic platform which can not only be used for *C. difficile, Strep B*, and *Streptococcus A*, but also other infections such as HSV 1&2, Mycoplasma, Pertussis, and CMV (Gene POC Technology, 2019). BioFire Film Array by BioMérieux is a multiplex PCR technology that has a wide range of panels to test for various disorders and pathogens^1^^,^^2^. GeneXpert systems by Cepheid is a near POCT NAAT that relies on hemi-nested qualitative PCR. GeneXpert HIV-1 Qual and GeneXpert MTB/RIF panels are currently being used for early infant HIV diagnosis and MTB/Rifampicin detection (Saeed et al., 2017; Opollo et al., 2018). With the ongoing COVID-19 pandemic, Xpert Xpress SARS-Cov2 test by Cepheid was the first to be approved by FDA for coronavirus testing and can produce results in about 45 min (Opollo et al., 2018). Nuclisens EasyQ by BioMérieux is an automated NASBA real time detection system that is used to monitor viral loading in HIV positive patients and provides results within 2 h (Tröger et al., 2015). ID Now System by Abbott Diagnostics is another isothermal amplification based rapid test that provides results within a few minutes for COVID-19, RSV, Strep A2 and Influenza A and B2^3^^,^^4^. Binx IO is the first FDA approved PCR amplification based rapid diagnostic test to detect sexually transmitted infections such as chlamydia and gonorrhea within 30 min^5^.

#### Advantages

NAAT assays can be fully automated and are a good solution for sample-in-answer-out testing in low resource settings (Niemz et al., 2011). Microfluidic based point-of-care nucleic acid testing is a sensitive, specific and an easy to use tool for disease detection as it requires a small volume of sample to amplify the target DNA/RNA (Lee et al., 2019).

#### Limitations/Challenges

Some of the limitations of/challenges with using NAAT based tests, are: (a.) Nucleic acid amplification using a conventional PCR requires complex & bulky equipment for thermal cycling, higher power consumption and longer turn-around times (Lee et al., 2019); (b.) Several non-PCR based isothermal amplification techniques such as rolling circle amplification (RCA), strand displacement amplification (SDA) and nucleic acid sequence based amplification (NASBA) still require high temperatures of 95°C to denature dsDNA (Lee et al., 2019); (c.) NAATs require additional steps of DNA/RNA extraction and purification which add to complexity and delay in results (Walker and Hsieh, 2019); (d.) The diagnostic performance of NAATs is dependent on the type of sample used and can be affected by several amplification inhibitors present in unprocessed samples (Al-Soud and Rådström, 2001; Sidstedt et al., 2018; Lee et al., 2019). (e.) Isothermal amplification techniques such as LAMP requires more than three primers therefore there is a high risk of primer dimer formation which can lead to false positives and undermine the accuracy of the POC test results (Becherer et al., 2020); (f.) Several isothermal NAAT technologies are still proprietary thereby posing a challenge to cost effectiveness as a POC (Kilic et al., 2020); (g.) Multiplex LAMP assays are still limited as compared to PCR (Sahoo et al., 2016).

## Other Microfluidic POCT Diagnostics Platforms

**Figure 5:**
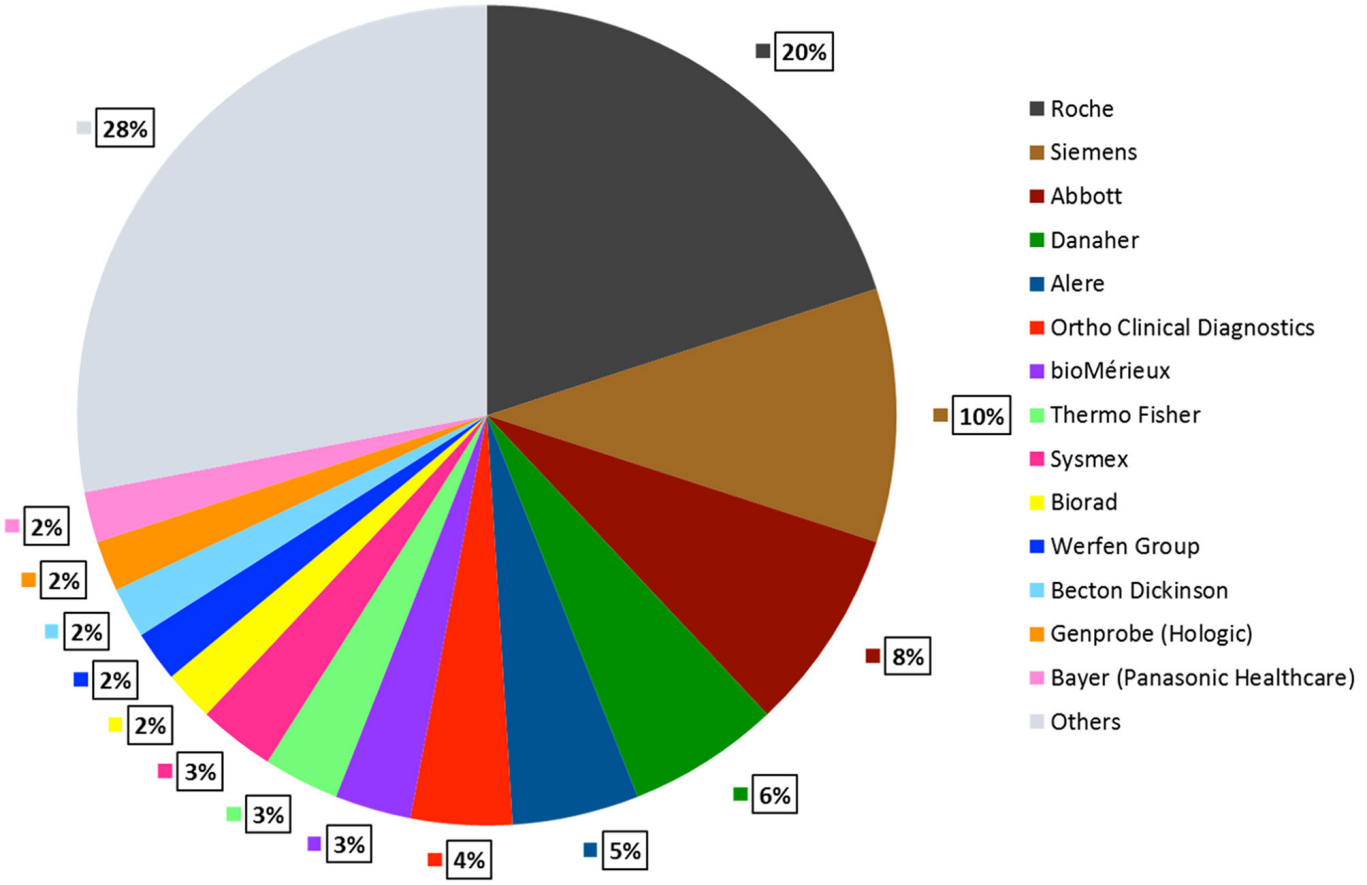
POCT Global Market Share by Company. Roche Diagnostics accounts for the largest market share of 20%, followed by Siemens 10%, Abbott 8%, Danaher 6%, Alere 5%, remaining companies contribute 2–4%. Other small and mid-sized companies hold 29% of the total market share (DUBLIN, 2018).

There are 14 top *in-vitro* diagnostic (IVD) companies controlling 70% of the market. Roche Diagnostics and Siemens Healthcare are tier I competitors that generate more than $5 Billion IVD revenues whereas companies such as Abbott, Danaher, Alere, Thermo Fisher, BioMérieux, Bio-Rad, Sysmex, Becton Dickinson, Bayer Healthcare, Werfen Group, Gen Probe generate $1–5 Billion revenues (DUBLIN, 2018). Figure 5 describes the global market share that these companies account for. In the previous sections, we illustrated some commercially available LFA and NAAT platforms. In this section, we discuss some popular microfluidic diagnostic devices from the aforementioned companies, based on technologies other than LFA and NAATs.

### iSTAT-System: Abbott

An easy to use, portable blood analyzer system introduced by Abbott, it utilizes disposable cartridge technology to provide rapid lab quality results within minutes. The cartridge consists of a silicon wafer with electrical sensors, a sample well, a waste chamber, a pouch with calibrant solutions. Cartridges range from lactate to cardiac markers to endocrinology. The wireless version of this device is called iSTAT1 Wireless^6^^,^^7^^,^^8^.

### Afinion System: Abbott

A cartridge-based analyzer used to monitor Diabetes and Albumin/Creatinine Ratio. The cartridges contain a sample collecting slot as well as the necessary reagents enabling automation to provide rapid quantitative results using urine or whole blood samples^9^. Afinion HbA1c Dx Test is a CLIA waived, automated boronate affinity test to quantify glycated hemoglobin. The analyzer uses a digital diode and a light emitting diode (LED) for reflection and transmission measurement^10^.

### HemoCue Hb201: Danaher

A handheld hemoglobin analyzer, it uses their patented microcuvette technology to quantify hemoglobin from whole blood. The microcuvette contains the dried reagents, which in contact with the blood lead to an azidemethemoglobin reaction. The HemoCue technique is based on optical measuring microcuvette of small volume and a short light path^11^.

### Xprecia Stride: Siemens

A rapid diagnostic device that uses electrochemical technology to quantify INR (International Normalized Ratio) based on a prothrombin time (PT) response in whole blood. The strip for the finger stick assay contains Dade & Innovin reagents which when combined with the sample, activate a coagulation cascade and the analyzer interprets the results^12^.

### Accu-Chek Aviva: Roche

An at-home blood-glucose monitoring system uses electrochemical technology to measure glucose in fresh whole blood. The glucose strips contain a modified Glucose dehydrogenase pyrroloquinoline quinone (GDH-PQQ) enzyme that converts glucose to gluconolactone; a DC current is generated, and the analyzer interprets the blood glucose value (Pashchenko et al., 2018).

### DCA Vantage System: Siemens

This is a rapid immunoassay analyzer glycemic index monitor which requires no sample or reagent prep. This CLIA waived HbA1c is a monoclonal agglutination reaction test, which provides results in 6 min. The immune cartridges contain the assay reagents and a sample well. Whole blood is sampled into the slot using a simple finger stick^13^. Urine is used for the Albumin/Creatinine ratio test^14^.

## COVID-19 Diagnostic Testing

Amidst the novel coronavirus (COVID-19) pandemic, the importance of rapid testing and early disease diagnosis has been underscored. Eliminating the need of highly skilled professionals and complicated protocols, point-of-care tests remain an urgent need to fight the active pandemic situation (Augustine et al., 2020; Joung et al., 2020). As of August 5, 2020, more than 18 million positive cases have been confirmed globally with the death toll reaching nearly 710 thousand (Pai et al., 2012). As of August 5, 2020, about 58,903,657 tests for COVID-19 have been performed in the US (Augustine et al., 2020). In this section, we discuss basic diagnostic testing for COVID-19 that are based on the microfluidic NAAT & LFA technology described in the previous sections of this article.

Currently most of the testing for COVID-19 require a trained healthcare professional and sophisticated equipment to collect and process the samples^15^. Even though FDA recently gave emergency use authorization to several at-home specimen collection kits – Pixel by LabCorp^16^, SARS-CoV-2 MALDI-TOF Assay by Ethos Laboratories^17^, KPMAS Covid-19 test by Kaiser Permanente Mid-Atlantic States^18^ – none of them have been approved by the FDA yet^19^. The basic workflow for such tests is using telemedicine, patients complete an online survey to check for eligibility to purchase the kit; once they have the kit, they collect the sample under the supervision of a healthcare provider; and ship the nasal swabs back to the lab for testing.

Two types of tests currently available for COVID-19 are:

A. *Diagnostic tests* – Check for an active infection in the body. These are further of two types:

i. Molecular Tests: Also called viral tests or nucleic acid amplification tests (NAATs), these are based on either RT-PCR or isothermal amplification (LAMP) technology to detect the RNA of novel coronavirus from a patient's respiratory sample^18^. Samples can be collected using a throat or a nasal swab. In some cases, Saliva is also used. Based on the current data, NAATs are highly accurate in diagnosing COVID-19, and used to check for current infection in the body^14, 18^.

ii. Antigen Tests: Also called rapid diagnostic tests, these detect specific proteins on the surface of the virus. Although this type of testing is faster in diagnosing an active COVID-19 infection as compared to molecular tests, there is a higher chance of it missing an active infection (*false negative*)^18^. Sofia 2 SARS Antigen FIA by Quidel Corporation is the first antigen to be developed and approved by FDA for emergency usage (Commissioner OOT, 2020). BD Veritor System for Rapid Detection of SARS-CoV-2 by Becton, Dickinson and Company, is another antigen detection test, which got EUA approval^20^.

B. *Serological Tests*: Also called antibody blood tests, these check for past infection by detecting antibodies our body may have developed in response to COVID-19^18^. Most of the tests detect IgM/IgG antibodies. However, the accuracy of antibody tests to diagnose coronavirus is still in question as they might not be able to indicate whether the patient developed the infection. In some cases, it may show a negative result due to no antibody formation even when the patient was infected (*false negative*) or the other way around (*false positive*). Therefore, in the current state serological testing is only helping healthcare workers better understand the immune response toward the coronavirus infection. Sample collecting id done using either a blood draw or a finger stick^18^,^21^^,^^22^.

### Commercially and Laboratory Developed Diagnostic Tests for COVID-19

As of August 8, 2020, more than 200 commercial manufacturers and laboratories that have received FDA EUA clearance. In this section, we discuss a few of these, including some rapid point-of-care based tests. Table 1 summarizes current tests available for COVID-19.

**Table 1:** List of commercial and laboratory developed COVID-19 diagnostic tests.

**Company name**	**Test name**	**Test type**	**Turn around time (TAT)**
Roche diagnostics	cobas^®^ SARS-CoV-2 test	Molecular	~ 3 h
Roche diagnostics	Elecsys^®^ Anti-SARS-CoV-2	Total antibody	18 min
Applied biosystems	TaqPath COVID-19 Combo Kit	Molecular	~ 3 h
Abbott molecular	Abbott RealTime SARS-C0V-2	Molecular	~ 24 h
Abbott diagnostics	ID NOW™ COVID-19	Molecular	5 Min
Abbott diagnostics	SARS-CoV-2 IgG assay	Antibody	N/A
Cephied	Xpert Xpress SARS-CoV-2	Molecular (NAAT)	30 min
MesaBiotech	Accula SARS-CoV-2	Molecular (NAAT)	30 Min
Cellex Inc.	qSARS-CoV-2	Antibody IgG/IgM (LFA)	15–20 min
Quidel corporation	Sofia 2 SARS Antigen FIA	Antibody	N/A
AccessBio	CareStart COVID-19 IgG/IgM	Antibody IgG/IgM (LFA)	10 min
Stanford health care	RT-PCR	Molecular	N/A
Rutgers's university	Saliva SARS-Cov-2 test	Molecular	N/A

Roche Diagnostics has developed both types of tests which have been cleared by the FDA for emergency usage. The cobas SARS-CoV-2 is a RT-PCR based NAAT which can handle 96 samples per assay whereas the Elecsys Anti-SARS-CoV-2 is an electrochemiluminescence immunoassay based^23^^,^^24^.

Applied Biosystems division of Thermo Fisher Scientific has developed TaqPath COVID-19 Combo kit, that contains assays and controls to detect COVID-19. This molecular diagnostic test targets orf1ab, spike protein and nucleocapsid protein genes and can facilitate 96 samples per assay^25^.

Abbott has launched three tests in response to COVID-19. Abbott Real Time SARS-CoV-2 assay was the first launched by Abbott Molecular that targets the RdRp and N-genes of the novel coronavirus. This test can handle up to 470 patient samples and provides results within 24 h^26^. ID Now COVID-19 is a CLIA waived, rapid, point-of-care isothermal amplification technology-based test by Abbott Diagnostics that can give positive test results for coronavirus within 5 min^27^. SARS-VoV-2 Immunoassay is the third test launched by Abbott Laboratories to detect IgG antibodies from serum and plasma samples using chemiluminescent microparticle immunoassay (CMIA) technology^28^.

Leveraging the GeneXpert technology, Cepheid launched Xpert Xpress SARS-CoV-2 molecular test which was the first point-of-care test to be approved by FDA for emergency use. The test can give results within 30 min (Opollo et al., 2018).

Mesa Biotech also received FDA emergency use authorization for its rapid point-of-care molecular test called Accula SARS-CoV-2, that uses nasal or throat swabs as samples and provides results within 30 min^29^.

Cellex Inc. has also developed a CLIA waived lateral flow technology based rapid test called qSARS-CoV-2 Rapid test, to detect IgM and IgG antibodies from serum or whole blood samples within 15–20 min (Etherington, 2020).

Quidel Corporation launched the first antigen test called Sofia 2 SARS Antigen FIA. It is a lateral flow sandwich assay that quantifies the nucleocapsid protein antigen from SARS-CoV-2 in nasopharyngeal (NP) and nasal (NS) swabs (Commissioner OOT, 2020).

AccessBio, Inc has launched a lateral flow assay based test called CareStart COVID-19 IgM/IgG that can detect IgG and IgM antibodies in response to COVID-19, and provide results in 10 min^30^.

Stanford Health Care and Clinical Laboratory developed their own RT-PCR molecular test, used to test patients at Stanford Health Care as well as Stanford Children's Care. This dual assay first screens for the presence of viral RNA by targeting the envelope protein and then confirms a positive result by targeting the RdRp gene^31^.

Rutger University laboratories, RUCDR Infinite Biologics and other collaborators recently got FDA EUA clearance for a saliva based diagnostic test for COVID-19 at-home sample collection^32^.

## Future Directions

The integration of microfluidics in point-of-care testing has significantly changed disease diagnosis and pathogen detection. Ease of use, no requirement of skilled personnel or heavy equipment, low sample volume, and rapid results have made POCT devices an indispensable part of the healthcare industry (Kankaanpää et al., 2018; Pezzuto et al., 2019). The POCT market is expected to reach USD 25.4 Million by 2022, expanding at a Compound Annual Growth Rate (CAGR) of 5.7% (DUBLIN, 2018). As previously discussed, some of the growing point-of-care testing products include blood glucose testing, infectious diseases testing, cardiac markers testing and coagulation testing. The key companies driving the POCT market are Abbott Laboratories, Danaher Corporation, Beckman Coulter Inc., Siemens AG and Abaxis (DUBLIN, 2018; Health Care, 2020; POCT Market Forecast, 2020). In this section, we discuss some emerging platforms that integrate microfluidics in point-of-care testing and may be a part of the next generation diagnostics.

### Smartphones and Wearable POCT

Advent of technology has led to the development of smartphone and wearable diagnostics. Using a sensor, mobile POCT detects signals from the sample *in vitro* whereas wearable POCT detects signals on the body. Both systems then send quantified results to the clinic via wireless communication. The technology on which these devices rely are either microfluidic or lateral flow based. Various body fluids such as tears, urine, blood, sweat, saliva can be used to analyze metabolites, hormones, proteins, viruses and bacteria (Shrivastava et al., 2020). Smartphones serve as minicomputers for sensitive and specific data quantification with built-in sensors, high resolution cameras, rapid wireless connectivity, ability to use many software's and apps and hence alone can be integrated as sensors and detectors in mobile POCT (Kanchi et al., 2018). mChip and dongles mimic immunoassays on a chip (Laksanasopin et al., 2015), whereas other smartphone-based devices detect malaria in microcapillary flow assays and test for changes in pH from saliva (Erickson et al., 2014). Enhanced biosensing technologies such as SAW, Nano sensing, SPR-surface plasmon resonance, development of new apps and cloud based data sharing, will shape the future of smartphone based devices for diagnosis (Turbé et al., 2017; Ghosh et al., 2020). Unlike smartphone based diagnostics, several components are needed to put together a wearable POCT (a.) sample handling component, (b.) sensing component, (c.) power component, (d.) signal detection and processing component and (e.) read out component (Shrivastava et al., 2020). Wearables can be physical sensors based to detect emotions, motion, heart rate, temperature or biochemical sensors-based samples from skin, eye or mouth with minimal invasion (Shrivastava et al., 2020). They come in different forms such as tattoos, patches, bands, watches, glasses, contact lenses, and can be integrated with smartphones for data requisition. This type of testing is especially important to people suffering from critical illness as they can monitor their health intermittently without the need of going to hospitals or needing trained personnel. Some examples of commercially available wearables include Glass by Google to monitor, prostate specific antigen (PSA), HIV and motor visual impairments (Feng et al., 2014; Dougherty and Badawy, 2017); and Guardian Sensor 3 by Medtronic^33^ and FreeStyle Libre by Abbott to monitor blood glucose levels (Yang and Gao, 2019).

### Emerging Microfluidic Start-Ups

Apart from diagnostics and point-of-care testing, other applications of microfluidic technology include agriculture, food safety, fertility and gamete research, single cell analysis and sequencing. Some of the pioneering microfluidic start-ups include:

10x Genomics is a fast-growing company based in California, USA focusing on single cell analysis with a high spending capacity. Their Next GEM technology is an amalgamation of droplet microfluidics and sequencing. In 2019, the company generated a total revenue of 245.9 M USD with a gross profit of 184.9M^34^.

Athelas Inc. is a rapidly expanding California based start-up founded in 2016. It has combined machine learning and lateral flow technology to develop a FDA approved at-home hematology analyzer and generates about 376K USD in sales. In 2019, the company raised a total of 20.6M USD^35^^,^^36^.

Butterfly iQ by Butterfly Network has revolutionized imaging by launching a cost-effective portable ultrasound device that uses a silicon chip technology instead of a transducer based whole body ultrasound scanner. Since it being founded in 2011, the growth trajectory of this Connecticut based company in the US has been high, with high spending capacity. The company has raised a total of 350 M USD with a post money valuation of 1.3B USD^37^^,^^38^.

Sandstone Diagnostics is another emerging healthcare start-up whose FDA approved Trak technology can provide rapid results of sperm count and semen volume at home. Torq zero acceleration centrifuge by Sandstone leverages microfluidic approach to provide blood samples and can easily replace a benchtop centrifuge especially in low resource settings. Since 2012, the company has raised 10.8 M USD and generates about 61 K USD in annual sales. In 2019, the company raised 2.5 M USD^39^^,^^40^.

Hesperos Inc. is a US based company that manufactures customized multi-organ chips to eliminate animal testing and enhance disease modeling. The company was founded in 2015 and has raised 4 M USD till date. The annual sales generate around 61 K USD^41^^,^^42^.

GenePOC, a Canadian company now part of Meridian Biosciences, uses automated cartridge based microfluidic technology for disease diagnosis and early detection. The company has a high growth trajectory and spending capacity. It has generated 4.9 M USD in annual sales till date^43^^,^^44^.

### Bottlenecks of Commercialization of Microfluidic POCT Platforms

Despite being around for a few decades, majority of lab-on-chip systems are yet to be commercialized or to be used routinely as research grade instruments outside specialized laboratories (Mohammed et al., 2015). Current POCT diagnostics are in the early development stage with limited multiplexing capacity & high system complexity (Dincer et al., 2017). There is also requirement for skilled technicians would be required for device operations and maintenance (Mohammed et al., 2015). Developing a new POCT requires significant capital investment and therefore, fundraising and the choice of market is another challenge that needs to be overcome in order to bring new technology into the market. With global competition and a constant time pressure, establishing a dominant position in the market can be time consuming as it requires lengthy clinical validation studies, complex regulatory approvals and slow clinical adoption (Chin et al., 2012). Another key aspect preventing promising technology to convert into a commercial product is the lack of standardization, which increases the complexity of manufacturing these devices making the entire manufacturing process labor intensive with increased production cost (Mohammed et al., 2015; Ghosh et al., 2019). Complexity in manufacturing & integrating several components together also thwart the process of large-scale production (Becker, 2009; Mohammed et al., 2015; Ghosh et al., 2019). There are several regulatory approvals required before a product can be introduced in the market, further increasing the time needed to be introduced in the market, and increasing the overall costs. Accuracy, sensitivity & specificity of a rapid test can also be a roadblock to its commercialization; FDA recently alerted the health care providers for false positive results with several SARS-CoV-2 antigen tests^45^. Lack of government support in cross-partnering also poses a challenge to diagnostic developers. For example, during the COVID-19 pandemic, several UK based companies were willing to manufacture other company tests in order to meet the demand and the need of the market. However, due to lack of government support, they were unable to do so^46^. Even in USA, there was lack of appropriate Government support in developing diagnostics for COVID-19 detection. Data digitization also increases the concerns about data privacy, compliance and patient data protection regulations, leading to high regulation scrutiny and litigation risks.

## Conclusion

In the past couple decades, point-of-care testing has seen sensational innovation. Microfluidic based POCT aims to provide rapid results to improve disease detection and diagnosis using small sample volumes. Although the accuracy and sensitivity of POCT as compared to conventional lab-based testing is still debatable, the COVID-19 pandemic accentuated the need for more rapid testing platforms. It will be interesting to see how future innovations in science and technology will bridge this gap, and if POCT will truly become the go-to for disease diagnosis.

## Author Contributions

SS: writing—original draft preparation, writing—reviewing, and editing. RD: writing—reviewing, editing, supervision, and secure funding. AS: conceptualization, writing—original draft preparation, writing—reviewing, editing, and supervision. All authors contributed to the article and approved the submitted version.

## Conflict of Interest

The authors declare that the research was conducted in the absence of any commercial or financial relationships that could be construed as a potential conflict of interest.

## Footnotes

^1^FilmArray Panels. Available online at: https://www.biofiredx.com/products/the-filmarray-panels/#blood-culture.

^2^Alethia Molecular Diagnostics. Available online at: https://www.meridianbioscience.com/platform/molecular/alethia.

^3^Nuclisens EasyQ. Available online at: https://www.biomerieux-diagnostics.com/nuclisens-easyqr.

^4^ID Now FDA. Available online at: https://www.fda.gov/media/136525/download.

^5^ID Now. Available online at: https://www.alere.com/en/home/product-details/id-now.html.

^6^Binx IO. Available online at: https://mybinxhealth.com/point-of-care/.

^7^iSTAT Corporation. Available online at: https://www.sec.gov/Archives/edgar/data/882365/000091205702038057/a2090815zex-99_1.htm#toc_ka8573_6.

^8^iSTAT Cartridges. Available online at: https://www.pointofcare.abbott/us/en/offerings/istat/istat-test-cartridges.

^9^iSTAT System, Abbott. Available online at: https://www.pointofcare.abbott/us/en/offerings/istat/istat-handheld#testing process.

^10^Afinion System FDA 1. Available online at: https://www.accessdata.fda.gov/cdrh_docs/reviews/K050574.pdf.

^11^Afinion Analyzer Technology. Available online at: https://www.stat-technologies.com/wp-content/uploads/2019/04/Afinion-2-Analyzer-User-Manual-.pdf.

^12^HemoCue Hb201 FDA.

^13^DCA ACR kit. Available online at: https://www.siemens-healthineers.com/en-us/diabetes/reagents/dca-microalbumin-creatinine-acr-urine.

^14^DCA Vantage System. Available online at: https://www.siemens-healthineers.com/en-us/diabetes.

^15^The COVID Tracking Project. Available online at: https://covidtracking.com/.

^16^Pixel, Labcor. Available online at: https://www.pixel.labcorp.com/at-home-test-kits/covid-19-test-home-collection-kit.

^17^LabCorp COVID-19. Available online at: https://www.fda.gov/media/136148/download.

^18^FAQ's Diagnostic Tests, CDC. Available online at: https://www.fda.gov/medical-devices/emergency-situations-medical-devices/faqs-testing-sars-cov-2.

^19^EUA, FDA. Available online at: https://www.fda.gov/medical-devices/coronavirus-disease-2019-covid-19-emergency-use-authorizations-medical-devices/vitro-diagnostics-euas.

^20^*Sofia 2 SARS Antigen Test*. FDA. Available online at: https://www.fda.gov/media/137886/download.

^21^Ethos Laboratories SARS-CoV-2 MALDI-TOF Assay. FDA. Available online at: https://www.fda.gov/media/140777/download.

^22^Testing for COVID-19 | CDC. Available online at: https://www.cdc.gov/coronavirus/2019-ncov/symptoms-testing/testing.html.

^23^COVID-19, Antigen Test. Available online at: https://www.fda.gov/news-events/press-announcements/coronavirus-covid-19-update-fda-authorizes-first-antigen-test-help-rapid-detection-virus-causes.

^24^Roche Diagnostics. Available online at: https://diagnostics.roche.com/global/en/c/covid-19-pandemic.html.

^25^Elecsys IL-6. Available online at: https://diagnostics.roche.com/us/en/products/params/elecsys-il-6.html.

^26^Applied Biosystems. Available online at: https://www.thermofisher.com/us/en/home/clinical/clinical-genomics/pathogen-detection-solutions/coronavirus-2019-ncov/genetic-analysis/taqpath-rt-pcr-covid-19-kit.html.

^27^How ID Now works.

^28^ID Now COVID-19.

^29^Mesa Biotech. Available online at: https://www.mesabiotech.com/coronavirus.

^30^Cellex Inc. Available from: https://cellexcovid.com/.

^31^*AccessBio Inc. CARESTART Covid-19*. Available from: https://www.fda.gov/media/140444/download.

^32^Rutger University, Saliva Test. Available online at: https://www.rutgers.edu/news/new-rutgers-saliva-test-coronavirus-gets-fda-approval.

^33^Google Glass. Available online at: https://www.google.com/glass/tech-specs/.

^34^10x Genomics. Available online at: https://www.10xgenomics.com/technology/.

^35^Company Information, 10x Genomics.

^36^Athelas, Company Profile 2. Available online at: https://craft.co/athelas.

^37^Butterfly's Ultrasound-on-a-chi. Available online at: https://www.med-technews.com/features/the-butterfly-effect-how-a-silicon-chip-is-easing-ultrasound/.

^38^Butterfly iQ - Ultrasound, ultra-simplified. Available online at: https://www.butterflynetwork.com/.

^39^Butterfly Networks. Available online at: https://app-avention-com.stanford.idm.oclc.org/company/f42f7829-f181-368f-9eb5-ac40426e3c7e#report/company_summary.

^40^Sandstone Diagnostics, Company Profile 2. Available online at: https://app-avention-com.stanford.idm.oclc.org/company/cfa224ae-ad40-30db-89f9-034c8122f3da#report/company_summary.

^41^Sandstone Diagnostics, Company Profile 1. Available online at: https://www.crunchbase.com/organization/sandstone-diagnostics#section-funding-rounds.

^42^Hesperos Inc Company Profile 2. Available online at: https://app-avention-com.stanford.idm.oclc.org/company/f20f8b3c-811d-306c-bd5f-b339ee4b8ac3#report/company_summary.

^43^Revogene Molecular Diagnostics. Available online at: https://www.meridianbioscience.com/platform/molecular/revogene/.

^44^Hesperos Inc Company Profile 1. Available online at: https://www.crunchbase.com/organization/hesperos#section-investors.

^45^*Potential for False Positive Results with Antigen Tests for Rapid Detection of SARS-CoV-2 - Letter to Clinical Laboratory Staff and Health Care Providers*. Available online at: https://www.fda.gov/medical-devices/letters-health-care-providers/potential-false-positive-results-antigen-tests-rapid-detection-sars-cov-2-letter-clinical-laboratory.

^46^*Developing Diagnostics: Challenges and Opportunities during COVID-19*. Available online at: https://www.med-technews.com/features/developing-diagnostics-challenges-and-opportunities-during-c/.

## References

[B1] Akyazi T. Basabe-Desmonts L. Benito-Lopez F. (2018). Review on microfluidic paper-based analytical devices towards commercialisation. Anal. Chim. Acta 1001, 1–17. 10.1016/j.aca.2017.11.010 29291790

[B2] Al-Soud W. A. Rådström P. (2001). Purification and characterization of PCR-inhibitory components in blood cells. J. Clin. Microbiol. 39, 485–493. 10.1128/JCM.39.2.485-493.2001 11158094 PMC87763

[B3] Augustine R. Hasan A. Das S. Ahmed R. Mori Y. Notomi T.. (2020). Loop-Mediated Isothermal Amplification (LAMP): a rapid, sensitive, specific, and cost-effective point-of-care test for coronaviruses in the context of COVID-19 pandemic. Biology 9:184. 10.3390/biology9080182 32707972 PMC7464797

[B4] Bahadir E. B. Sezgintürk M. K. (2016). Lateral flow assays: principles, designs and labels. TrAC Trends Analy. Chem. 82, 286–306. 10.1016/j.trac.2016.06.006

[B5] Ballard Z. Ozcan A. (2018). Nucleic acid quantification in the field. Nat. Biomed. Eng. 2, 629–630. 10.1038/s41551-018-0292-0 31015678

[B6] Becherer L. Borst N. Bakheit M. Frischmann S. Zengerle R. von Stetten F. (2020). Loop-mediated isothermal amplification (LAMP) – review and classification of methods for sequence-specific detection. Analy. Methods 12, 717–746. 10.1039/C9AY02246E

[B7] Becker H. (2009). It's the economy. Lab. Chip 9, 2759–2762. 10.1039/b916505n 19967108

[B8] Bhandari P. Narahari T. Dendukuri D. (2011). 'Fab-chips': a versatile, fabric-based platform for low-cost, rapid and multiplexed diagnostics. Lab Chip 11, 2493–2499. 10.1039/c1lc20373h 21735030

[B9] Caetano F. R. Carneiro E. A. Agustini D. Figueiredo-Filho L. C. S. Banks C. E. Bergamini M. F.. (2018). Combination of electrochemical biosensor and textile threads: a microfluidic device for phenol determination in tap water. Biosens. Bioelectron. 99, 382–388. 10.1016/j.bios.2017.07.070 28806668

[B10] Carrio A. Sampedro C. Sanchez-Lopez J. L. Pimienta M. (2015). Campoy automated low-cost smartphone-based lateral flow saliva test reader for drugs-of-abuse detection. Sensors 15, 29569–29593. 10.3390/s151129569 26610513 PMC4701349

[B11] Chen J. Zhou Y. Wang D. He F. Rotello V. M. Carter K. R.. (2015). UV-nanoimprint lithography as a tool to develop flexible microfluidic devices for electrochemical detection. Lab Chip 15, 3086–3094. 10.1039/C5LC00515A 26095586

[B12] Chiappin S. Antonelli G. Gatti R. De Palo E. F. (2007). Saliva specimen: a new laboratory tool for diagnostic and basic investigation. Clin. Chim. Acta 383, 30–40. 10.1016/j.cca.2007.04.011 17512510

[B13] Chin C. D. Linder V. Sia S. K. (2012). Commercialization of microfluidic point-of-care diagnostic devices. Lab Chip 12, 2118–34. 10.1039/c2lc21204h 22344520

[B14] Commissioner OOT (2020). Coronavirus Disease 2019 (COVID-19) Frequently Asked Questions. Gwalior: FDA.

[B15] Cox C. R. Jensen K. R. Mondesire R. R. Voorhees K. J. (2015). Rapid detection of *Bacillus anthracis* by γ phage amplification and lateral flow immunochromatography. J. Microbiol. Methods 118, 51–56. 10.1016/j.mimet.2015.08.011 26310605

[B16] Craw P. Balachandran W. (2012). Isothermal nucleic acid amplification technologies for point-of-care diagnostics: a critical review. Lab Chip 12, 2469–2486. 10.1039/c2lc40100b 22592150

[B17] de Puig H. Bosch I. Gehrke L. Hamad-Schifferli K. (2017). Challenges of the nano–bio interface in lateral flow and dipstick immunoassays. Trends Biotechnol. 35, 1169–1180. 10.1016/j.tibtech.2017.09.001 28965747 PMC5696013

[B18] Dincer C. Bruch R. Kling A. Dittrich P. S. Urban G. A. (2017). Multiplexed point-of-care testing – xPOCT. Trends Biotechnol. 35, 728–742. 10.1016/j.tibtech.2017.03.013 28456344 PMC5538621

[B19] Ding X. Wu W. Zhu Q. Zhang T. Jin W. Mu Y. (2015). Mixed-dye-based label-free and sensitive dual fluorescence for the product detection of nucleic acid isothermal multiple-self-matching-initiated amplification. Analy. Chem. 87, 10306–10314. 10.1021/acs.analchem.5b02112 26383158

[B20] Dougherty B. Badawy S. M. (2017). Using google glass in nonsurgical medical settings: systematic review. JMIR Mhealth Uhealth 5:e159. 10.2196/mhealth.8671 29051136 PMC5668637

[B21] DUBLIN (2018). In Vitro Diagnostics: Technologies and Global Markets in M2 Presswire. Coventry: Normans Media Ltd.

[B22] Erickson D. O'Dell D. Jiang L. Oncescu V. Gumus A. Lee S.. (2014). Smartphone technology can be transformative to the deployment of lab-on-chip diagnostics. Lab Chip 14, 3159–3164. 10.1039/C4LC00142G 24700127 PMC4117816

[B23] Estrela P. Katarzyna Koczula M. Gallotta A. (2016). Lateral flow assays. Essays Biochem. 60, 111–120. 10.1042/EBC20150012 27365041 PMC4986465

[B24] Etherington D. (2020). Mesa Biotech Gains Emergency FDA Approval for Rapid, Point-of-care COVID-19 Test. TechCrunch Available online at: https://techcrunch.com/2020/03/24/mesa-biotech-gains-emergency-fda-approval-for-rapid-point-of-care-covid-19-test/ (accessed August 8, 2020).

[B25] Feng S. Caire R. Cortazar B. Turan M. Wong A. Ozcan A. (2014). Immunochromatographic diagnostic test analysis using google glass. ACS Nano 8, 3069–3079. 10.1021/nn500614k 24571349 PMC3988681

[B26] Gene POC Technology. (2019). Available online at: https://www.genepoc-diagnostics.com/technology/

[B27] Gerald J. K. William J. F. Laurie E. K. (2014). Principles of point of care culture, the spatial care path, and enabling community and global resilience: enabling community and global resilience. EJIFCC 25, 134–153. 27683461 PMC4975289

[B28] Gessler F. Pagel-Wieder S. Avondet M-A. Böhnel H. (2007). Evaluation of lateral flow assays for the detection of botulinum neurotoxin type A and their application in laboratory diagnosis of botulism. Diagn. Microbiol. Infect. Dis. 57, 243–249. 10.1016/j.diagmicrobio.2006.07.017 17141460

[B29] Ghosh R. Gopalakrishnan S. Savitha R. Renganathan T. Pushpavanam S. (2019). Fabrication of laser printed microfluidic paper-based analytical devices (LP-μPADs) for point-of-care applications. Sci. Rep. 9:7896. 10.1038/s41598-019-44455-1 31133720 PMC6536539

[B30] Ghosh S. Aggarwal K. Vinitha T. U. Nguyen T. Han J. Ahn C. H. (2020). A new microchannel capillary flow assay (MCFA) platform with lyophilized chemiluminescence reagents for a smartphone-based POCT detecting malaria. Microsyst. Nanoeng. 6:5 10.1038/s41378-019-0108-8 PMC8433401 34567620

[B31] Haushalter K. J. Vetcha S. Haushalter R. C. (2016). Multiplex flow assays. ACS Omega 1, 586–599. 10.1021/acsomega.6b00188 27819063 PMC5088464

[B32] Health Care. (2020). New Rutgers Saliva Test for Coronavirus Gets FDA, and Approval. Rutgers University Available online at: https://www.rutgers.edu/news/new-rutgers-saliva-test-coronavirus-gets-fda-approval (accessed August 8, 2020)

[B33] Hristov D. R. Rodriguez-Quijada C. Gomez-Marquez J. Hamad-Schifferli K. (2019). Designing paper-based immunoassays for biomedical applications. Sensors 19:554. 10.3390/s19030554 30699964 PMC6387326

[B34] Hsu H.-L. Chuang C.-C. Liang C.-C. Chiao D.-J. Wu H.-L. Wu Y.-P.. (2018). Rapid and sensitive detection of Yersinia pestis by lateral-flow assay in simulated clinical samples. BMC Infect. Dis. 18:402. 10.1186/s12879-018-3315-2 30107826 PMC6092852

[B35] Hu J. Choi J. R. Wang S. Gong Y. Feng S. Pingguan-Murphy B. (2017). Multiple test zones for improved detection performance in lateral flow assays. Sens. Actuators B Chem. 243, 484–488. 10.1016/j.snb.2016.12.008

[B36] Joung J. Ladha A. Saito M. Segel M. Bruneau R. Huang M. W. (2020). Point-of-care testing for COVID-19 using SHERLOCK diagnostics. medRxiv. 10.1101/2020.05.04.20091231

[B37] Kanchi S. Sabela M. I. Mdluli P. S. Inamuddin Bisetty K. (2018). Smartphone based bioanalytical and diagnosis applications: a review. *Biosens*. Bioelectron. 102, 136–149. 10.1016/j.bios.2017.11.021 29128716

[B38] Kankaanpää M. Holma-Eriksson M. Kapanen S. Heitto M. Bergström S. Muukkonen L.. (2018). Comparison of the use of comprehensive point-of-care test panel to conventional laboratory process in emergency department. BMC Emerg. Med. 18:43. 10.1186/s12873-018-0198-x 30453888 PMC6245706

[B39] Kanter J. Telen M. J. Hoppe C. Roberts C. L. Kim J. S. Yang X. (2015). Validation of a novel point of care testing device for sickle cell disease. BMC Med. 13:225. 10.1186/s12916-015-0473-6 26377572 PMC4573998

[B40] Kaur H. Chaterjee B. Bruno J. G. Sharma T. K. (2019). Defining Target Product Profiles (TPPs) for aptamer-based diagnostics. Adv. Biochem. Eng. Biotechnol. 174, 195–209. 10.1007/10_2019_104 31332450

[B41] Kilic T. Weissleder R. Lee H. (2020). Molecular and immunological diagnostic tests of COVID-19: current status and challenges. iScience 23:101406. 10.1016/j.isci.2020.101406 32771976 PMC7381402

[B42] Kiran R. M. Chakraborty S. (2020). PDMS microfluidics: a mini review. J. Appl. Polymer Sci. 137:48958 10.1002/app.48958

[B43] Kokkinos C. Economou A. Speliotis T. Petrou P. Kakabakos S. (2015). Flexible microfabricated film sensors for the *in situ* quantum dot-based voltammetric detection of DNA hybridization in microwells. Analy. Chem. 87, 853–857. 10.1021/ac503791j 25514352

[B44] Laksanasopin T. Guo T. W. Nayak S. Sridhara A. A. Xie S. Olowookere O. O.. (2015). A smartphone dongle for diagnosis of infectious diseases at the point of care. Sci. Trans. Med. 7:273re1. 10.1126/scitranslmed.aaa0056 25653222

[B45] Lee S. H. Park S. M. Kim B. N. Kwon O. S. Rho W. Y. Jun B. H. (2019). Emerging ultrafast nucleic acid amplification technologies for next-generation molecular diagnostics. Biosens. Bioelectron. 141, 111448. 10.1016/j.bios.2019.111448 31252258

[B46] Lee-Lewandrowski E. Corboy D. Lewandrowski K. Sinclair J. McDermot S. Benzer T. I. (2003). Implementation of a point-of-care satellite laboratory in the emergency department of an academic medical center. impact on test turnaround time and patient emergency department length of stay. Arch. Pathol. Lab. Med. 127, 456–460. 10.1043/0003-9985(2003)127<0456:IOAPSL>2.0.CO;2 12683874

[B47] Li J. Macdonald J. (2016). Multiplexed lateral flow biosensors: technological advances for radically improving point-of-care diagnoses. Biosens. Bioelectron. 83, 177–192. 10.1016/j.bios.2016.04.021 27125840

[B48] Li X. Ballerini D. R. Shen W. (2012). A perspective on paper-based microfluidics: current status and future trends. Biomicrofluidics 6, 11301–1130113. 10.1063/1.3687398 22662067 PMC3365319

[B49] Li X. Tian J. Shen W. (2010). Thread as a versatile material for low-cost microfluidic diagnostics. ACS Appl. Mater. Interfaces 2, 1–6. 10.1021/am9006148 20356211

[B50] Luppa P. B. Müller C. Schlichtiger A. Schlebusch H. (2011). Point-of-care testing (POCT): current techniques and future perspectives. TrAC Trends Analy. Chem. 30, 887–898. 10.1016/j.trac.2011.01.019 32287536 PMC7125710

[B51] Magambo K. A. Kalluvya S. E. Kapoor S. W. Seni J. Chofle A. A. Fitzgerald D. W.. (2014). Utility of urine and serum lateral flow assays to determine the prevalence and predictors of cryptococcal antigenemia in HIV-positive outpatients beginning antiretroviral therapy in Mwanza, Tanzania. J. Int. AIDS Soc. 17:19040. 10.7448/IAS.17.1.19040 25109284 PMC4127809

[B52] Mahmoudi T. de la Guardia M. Baradaran B. (2020). Lateral flow assays towards point-of-care cancer detection: a review of current progress and future trend*s*. TrAC Trends Analy. Chem. 125:115842 10.1016/j.trac.2020.115842

[B53] Mahmoudi T. de la Guardia M. Shirdel B. Mokhtarzadeh A. Baradaran B. (2019). Recent advancements in structural improvements of lateral flow assays towards point-of-care testing. TrAC Trends Analy. Chem. 116, 13–30. 10.1016/j.trac.2019.04.016

[B54] Martinez A. W. Phillips S. T. Butte M. J. Whitesides G. M. (2007). Patterned paper as a platform for inexpensive, low-volume, portable bioassays. Angew. Chem. 46, 1318–1320. 10.1002/anie.200603817 17211899 PMC3804133

[B55] McDonald J. C. Duffy D. C. Anderson J. R. Chiu D. T. Wu H. Schueller O. J. A.. (2000). Fabrication of microfluidic systems in poly(dimethylsiloxane). Electrophoresis 21, 27–40. 10.1002/(SICI)1522-2683(20000101)21:1<27::AID-ELPS27>3.0.CO;2-C 10634468

[B56] Mohammed M. I. Haswell S. Gibson I. (2015). Lab-on-a-chip or chip-in-a-lab: challenges of commercialization lost in translation. Proc. Technol. 20, 54–59. 10.1016/j.protcy.2015.07.01032098268

[B57] Mohd Hanafiah K. Arifin N. Bustami Y. Noordin R. Garcia M. Anderson D. (2017). Development of multiplexed infectious disease lateral flow assays: challenges and opportunities. Diagnostics 7:51. 10.3390/diagnostics7030051 28880218 PMC5617951

[B58] Moreno M. L. Cebolla Á. Muñoz-Suano A. Carrillo-Carrion C. Comino I. Pizarro Á.. (2017). Detection of gluten immunogenic peptides in the urine of patients with coeliac disease reveals transgressions in the gluten-free diet and incomplete mucosal healing. Gut 66, 250–257. 10.1136/gutjnl-2015-310148 26608460 PMC5284479

[B59] Müller R. H. Clegg D. L. (1949). Automatic paper chromatography. Analy. Chem. 21, 1123–1125. 10.1021/ac60033a032

[B60] Naeimirad M. Abuzade R. Babaahmadi V. Dabirian F. (2019). Microfluidic through fibrous structures: recent developments and future trends. Mater. Design Process. Commun. 1:e78 10.1002/mdp2.78

[B61] Ndlovu Z. Massaquoi L. Bangwen N. E. Batumba J. N. Bora R. U. Mbuaya J.. (2020). Diagnostic performance and usability of the VISITECT CD4 semi-quantitative test for advanced HIV disease screening. PLoS ONE 15:e0230453. 10.1371/journal.pone.0230453 32243435 PMC7122771

[B62] Niemz A. Ferguson T. M. Boyle D. S. (2011). Point-of-care nucleic acid testing for infectious diseases. Trends Biotechnol. 29, 240–250. 10.1016/j.tibtech.2011.01.007 21377748 PMC3746968

[B63] Nilghaz A. Ballerini D. R. Shen W. (2013). Exploration of microfluidic devices based on multi-filament threads and textiles: a review. Biomicrofluidics 7:051501. 10.1063/1.4820413 24086179 PMC3779262

[B64] Opollo V. S. Nikuze A. Ben-Farhat J. Anyango E. Humwa F. Oyaro B.. (2018). Field evaluation of near point of care Cepheid GeneXpert HIV-1 qual for early infant diagnosis. PLoS ONE 13:e0209778. 10.1371/journal.pone.0209778 30589900 PMC6307732

[B65] Pai N. P. Vadnais C. Denkinger C. Engel N. Pai M. (2012). Point-of-care testing for infectious diseases: diversity, complexity, and barriers in low- and middle-income countries. PLoS Med. 9:e1001306. 10.1371/journal.pmed.1001306 22973183 PMC3433407

[B66] Pandey C. M. Augustine S. Kumar S. Kumar S. Nara S. Srivastava S.. (2018). Microfluidics based point-of-care diagnostics. Biotechnol. J. 13:1700047. 10.1002/biot.201700047 29178532

[B67] Parida MM Dash PK Shukla J. (2020). Advance detection technologies for select biothreat agents, in Handbook on Biological Warfare Preparedness, eds Flora S. J. S. Pachauri V. (Gwalior: Academic Press), 83–102.

[B68] Pashchenko O. Shelby T. Banerjee T. Santra S. (2018). A comparison of optical, electrochemical, magnetic, and colorimetric point-of-care biosensors for infectious disease diagnosis. ACS Infect. Dis. 4, 1162–1178. 10.1021/acsinfecdis.8b00023 29860830 PMC6736529

[B69] Pezzuto F. Scarano A. Marini C. Rossi G. Stocchi R. Di Cerbo A (2019). Assessing reliability of commercially available point of care in various clinical fields. Open Public Health J. 12, 342–368. 10.2174/1874944501912010342

[B70] Pillai S. P. DePalma L. Prentice K. W. Ramage J. G. Chapman C. Sarwar J.. (2020). Comprehensive laboratory evaluation of a specific lateral flow assay for the presumptive identification of *francisella tularensis* in suspicious white powders and aerosol samples. Health Secur. 18, 83–95. 10.1089/hs.2019.0151 32324068 PMC7194312

[B71] POCT Market Forecast. (2020). Available online at: https://www.grandviewresearch.com/press-release/global-point-of-care-diagnostics-market (accessed August 8, 2020)

[B72] Qamar A. Z. Parker G. Kinsel G. R. Shamsi M. H. (2019). Evolution of wax-on-plastic microfluidics for sub-microliter flow dynamics and its application in distance-based assay. Microfluidics Nanofluidics 23:81 10.1007/s10404-019-2249-3

[B73] Qamar A. Z. Shamsi M. H. (2020). Desktop fabrication of lab-on-chip devices on flexible substrates: a brief review. Micromachines 11:126. 10.3390/mi11020126 31979275 PMC7074936

[B74] Reches M. Mirica K. A. Dasgupta R. Dickey M. D. Butte M. J. Whitesides G. M. (2010). Thread as a matrix for biomedical assays. ACS Appl. Mater. Interfaces 2, 1722–1728. 10.1021/am1002266 20496913

[B75] Reid R. Chatterjee B. Das S. J. Ghosh S. Sharma T. K. (2020). Application of aptamers as molecular recognition elements in lateral flow assays. Analy. Biochem. 593:113574. 10.1016/j.ab.2020.113574 31911046

[B76] Ricciardi A. Crescitelli A. Vaiano P. Quero G. Consales M. Pisco M.. (2015). Lab-on-fiber technology: a new vision for chemical and biological sensing. Analyst 140, 8068–8079. 10.1039/C5AN01241D 26514109

[B77] Rumaner M. Horowitz L. Ovadya A. Folch A. (2019). Thread as a low-cost material for microfluidic assays on intact tumor slices. Micromachines 10:481. 10.3390/mi10070481 31319620 PMC6680473

[B78] Saeed M. Ahmad M. Iram S. Riaz S. Akhtar M. Aslam M. (2017). GeneXpert technology: A breakthrough for the diagnosis of tuberculous pericarditis and pleuritis in less than 2 hours. Saudi Med. J. 38, 699–705. 10.15537/smj.2017.7.17694 28674714 PMC5556276

[B79] Safavieh R. Zhou G. Z. Juncker D. (2011). Microfluidics made of yarns and knots: from fundamental properties to simple networks and operations. Lab Chip 11, 2618–2624. 10.1039/c1lc20336c 21677945

[B80] Saha A. K. Dallo S. F. Detmar A. L. Osmulski P. Gaczynska M. Huang T. H.. (2017a). Cellular cholesterol regulates monocyte deformation. J. Biomech. 52, 83–88. 10.1016/j.jbiomech.2016.12.033 28082022 PMC5736503

[B81] Saha A. K. Osmulski P. Dallo S. F. Gaczynska M. Huang T. H. Ramasubramanian A. K. (2017b). Cholesterol regulates monocyte rolling through cd44 distribution. Biophys. J. 112, 1481–1488. 10.1016/j.bpj.2017.02.021 28402890 PMC5389964

[B82] Saha A. K. Schmidt B. R. Wilhelmy J. Nguyen V. Abugherir A. Do J. K.. (2019). Red blood cell deformability is diminished in patients with chronic fatigue syndrome. Clin. Hemorheol. Microcirc. 71, 113–116. 10.3233/CH-180469 30594919 PMC6398549

[B83] Sahoo P. R. Sethy K. Mohapatra S. Panda D. (2016). Loop mediated isothermal amplification: an innovative gene amplification technique for animal diseases. Vet. World 9, 465–469. 10.14202/vetworld.2016.465-469 27284221 PMC4893716

[B84] Schramm E. C. Staten N. R. Zhang Z. Bruce S. S. Kellner C. Atkinson J. P.. (2015). A quantitative lateral flow assay to detect complement activation in blood. Analy. Biochem. 477, 78–85. 10.1016/j.ab.2015.01.024 25660530 PMC4404182

[B85] Shrivastava S. Quang Trung T. Lee N.-E. (2020). Recent progress, challenges, and prospects of fully integrated mobile and wearable point-of-care testing systems for self-testing. Chem. Soc. Rev. 49:1812. 10.1039/C9CS00319C 32100760

[B86] Sidstedt M. Hedman J. Romsos E. L. Waitara L. Wadsö L. Steffen C. R.. (2018). Inhibition mechanisms of hemoglobin, immunoglobulin G, and whole blood in digital and real-time PCR. Analy. Bioanaly. Chem. 410, 2569–2583. 10.1007/s00216-018-0931-z 29504082 PMC5857286

[B87] Singer J. M. Plotz C. M. (1956). The latex fixation test* I application to the serologic diagnosis of rheumatoid arthritis. Am. J. Med. 21, 888–892. 10.1016/0002-9343(56)90103-6 13372565

[B88] Su W. Gao X. Jiang L. Qin J. (2015). Microfluidic platform towards point-of-care diagnostics in infectious diseases. J. Chromatogr. A 13–26. 10.1016/j.chroma.2014.12.041 25544727

[B89] Sun J. Xianyu Y. Jiang X. (2014). Point-of-care biochemical assays using gold nanoparticle-implemented microfluidics. Chem. Soc. Rev. 43, 6239–6253. 10.1039/C4CS00125G 24882068

[B90] Tang R. Yang H. Gong Y. Liu Z. Li X. Wen T.. (2017). Improved analytical sensitivity of lateral flow assay using sponge for HBV nucleic acid detection. Sci. Rep. 7:1360. 10.1038/s41598-017-01558-x 28465588 PMC5431006

[B91] Tay A. Pavesi A. Yazdi S. R. Lim C. T. Warkiani M. E. (2016). Advances in microfluidics in combating infectious *diseases*. Biotechnol. Adv. 34, 404–421. 10.1016/j.biotechadv.2016.02.002 26854743 PMC7125941

[B92] Tröger V. Niemann K. Gärtig C. Kuhlmeier D. (2015). Isothermal amplification and quantification of nucleic acids and its use in microsystems. J. Nanomed. Nanotechnol. 6:3 10.4172/2157-7439.1000282

[B93] Tsaloglou M. N. Watson R. J. Rushworth C. M. Zhao Y. Niu X. Sutton J. M. (2015). Real-time microfluidic recombinase polymerase amplification for the toxin B gene of clostridium difficile on a slip chip platform. Analyst 140, 258–264. 10.1039/C4AN01683A 25371968

[B94] Turbé V. Gray E. R. Lawson V. E. Nastouli E. Brookes J. C. Weiss R. A.. (2017). Towards an ultra-rapid smartphone- connected test for infectious diseases. Sci. Rep. 7:11971. 10.1038/s41598-017-11887-6 28931860 PMC5607310

[B95] Tur-García E. L. Davis F. Collyer S. D. Holmes J. L. Barr H. Higson S. P. J. (2017). Novel flexible enzyme laminate-based sensor for analysis of lactate in sweat. Sens. Actuators B Chem. 242, 502–510. 10.1016/j.snb.2016.11.040

[B96] Urdea M. Penny L. A. Olmsted S. S. Giovanni M. Y. Kaspar P. Shepherd A.. (2006). Requirements for high impact diagnostics in the developing world. Nature 444, 73–79. 10.1038/nature05448 17159896

[B97] Walker F. M. Hsieh K. (2019). Advances in directly amplifying nucleic acids from complex samples. Biosensors 9:117. 10.3390/bios9040117 31574959 PMC6955841

[B98] Wang C. Peng J. Liu D.-F. Xing K.-Y. Zhang G.-G. Huang Z.. (2018). Lateral flow immunoassay integrated with competitive and sandwich models for the detection of aflatoxin M1 and Escherichia coli O157:H7 in milk. J. Dairy Sci. 101, 8767–8777. 10.3168/jds.2018-14655 30100502

[B99] Wang S. Ge L. Song X. Yu J. Ge S. Huang J.. (2012). Paper-based chemiluminescence ELISA: lab-on-paper based on chitosan modified paper device and wax*-*screen-printing. Biosens. Bioelectron. 31, 212–218. 10.1016/j.bios.2011.10.019 22051546

[B100] Weigl B. Domingo G. Labarre P. Gerlach J. (2008). Towards non-and minimally instrumented, microfluidics-based diagnostic devices. Lab Chip 8, 1999–2014 10.1039/b811314a 19023463 PMC2776042

[B101] Weltin A. Kieninger J. Enderle B. Gellner A.-K. Fritsch B. Urban G. A. (2014). Polymer-based, flexible glutamate and lactate microsensors for *in vivo* applications. Biosens. Bioelectron. 61, 192–199. 10.1016/j.bios.2014.05.014 24880657

[B102] Xing S. Jiang J. Pan T. (2013). Interfacial microfluidic transport on micropatterned superhydrophobic textile. Lab Chip 13, 1937–1947. 10.1039/c3lc41255e 23536189

[B103] Xu Y. Liu Y. Wu Y. Xia X. Liao Y. Li Q. (2014). Fluorescent probe-based lateral flow assay for multiplex nucleic acid detection. Analy. Chem. 86, 5611–5614. 10.1021/ac5010458 24892496

[B104] Yan C. Cui J. Huang L. Du B. Chen L. Xue G.. (2020). Rapid and visual detection of 2019 novel coronavirus (SARS-CoV-2) by a reverse transcription loop-mediated isothermal amplification assay. Clin. Microbiol. Infect. 26, 773–779. 10.1016/j.cmi.2020.04.001 32276116 PMC7144850

[B105] Yang Y. Gao W. (2019). Wearable and flexible electronics for continuous molecular monitoring. Chem. Soc. Rev. 48, 1465–1491. 10.1039/C7CS00730B 29611861

[B106] Yen C.-W. de Puig H. Tam J. O. Gómez-Márquez J. Bosch I. Hamad-Schifferli K.. (2015). Multicolored silver nanoparticles for multiplexed disease diagnostics: distinguishing dengue, yellow fever, Ebola viruses. Lab Chip 15, 1638–1641. 10.1039/C5LC00055F 25672590 PMC4375736

[B107] Yetisen A. K. Akram M. S. Lowe C. R. (2013). Paper-based microfluidic point-of-care diagnostic devices. Lab Chip 13, 2210–2251. 10.1039/c3lc50169h 23652632

[B108] Yew C.-H. T. Azari P. Choi J. R. Li F. Pingguan-Murphy B. (2018). Electrospin-coating of nitrocellulose membrane enhances sensitivity in nucleic acid-based lateral flow assay. Anal. Chim. Acta 1009, 81–88. 10.1016/j.aca.2018.01.016 29422135

